# Intratracheal instillation for the testing of pulmonary toxicity in mice—Effects of instillation devices and feed type on inflammation

**DOI:** 10.1002/ame2.12503

**Published:** 2025-01-03

**Authors:** Niels Hadrup, Michael Guldbrandsen, Eva Terrida, Katja M. S. Bendtsen, Karin S. Hougaard, Nicklas R. Jacobsen, Ulla Vogel

**Affiliations:** ^1^ National Research Centre for the Working Environment Copenhagen Denmark; ^2^ Research Group for Risk‐Benefit, National Food Institute Technical University of Denmark Copenhagen Denmark; ^3^ Department of Public Health University of Copenhagen Copenhagen Denmark; ^4^ National Food Institute Technical University of Denmark Copenhagen Denmark

**Keywords:** inhalation, mouse, pulmonary, toxicity

## Abstract

**Background:**

Inhalation exposure is the gold standard when assessing pulmonary toxicity. However, it typically requires substantial amounts of test material. Intratracheal instillation is an alternative administration technique, where the test substance is suspended in a liquid vehicle and deposited into the lung via the trachea. Instillation requires minimal test material, delivers an exact dose deep into the lung, and is less labor‐intensive than inhalation exposures. However, one shortcoming is that the procedure may induce short‐term inflammation. To minimize this, we tested different modifications of the technique to identify the potential for refinement.

**Methods:**

First, we tested whether previous findings of increased inflammation could be confirmed. Next, we tested whether instillation with a disposable 1 mL syringe with ball‐tipped steel‐needle (Disposable‐syringe/steel‐needle) induced less inflammation than the use of our standard set‐up, a 250 μL reusable glass syringe with a disposable plastic catheter (Glass‐syringe/plastic‐catheter). Finally, we tested if access to pelleted and liquid feed prior to instillation affected inflammation. We evaluated inflammation by neutrophil numbers in bronchoalveolar fluid 24 h post‐exposure.

**Results:**

Vehicle‐instilled mice showed a small increase in neutrophil numbers compared to untreated mice. Neutrophil numbers were slightly elevated in the groups instilled with Disposable‐syringe/steel‐needle; an interaction with feed type indicated that the increase in neutrophils was more pronounced in combination with feed pellets compared to liquid feed. We found no difference between the feed types when using the Glass‐syringe/plastic‐catheter combination.

**Conclusion:**

The Glass‐syringe/plastic‐catheter combination induced the least exposure‐related inflammation, confirming this as a preferred instillation procedure.

## Graphical abstract

Intratracheal instillation is a pulmonary administration technique in mice. We tested different modifications of intratracheal instillation to identify potential options for refinement. A reusable glass syringe with an Insyte plastic catheter caused less inflammation than a disposable syringe with ball‐tipped steel‐needle. There was no advantage in using liquid feed over feed pellets.

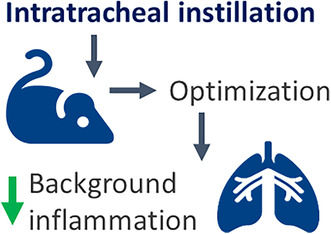

## 1 INTRODUCTION

When evaluating toxicity following pulmonary deposition of chemicals or particulates, inhalation is the natural route of entry and the gold standard for toxicity testing. However, intratracheal instillation is an alternative to inhalation in pulmonary exposure studies and poses some advantages, making this an interesting alternative. The technique was already used in humans at the end of the 19th century for the treatment of pulmonary inflammatory lesions with liquid petrolatum.^1^ As an experimental administration route in mice, the technique seems to have been introduced in the 1970s.^2^, ^3^ One advantage with intratracheal instillation is that it allows full control over the deposited dose. This is an advantage when comparing the toxicological response of different materials, since pulmonary deposition during inhalation may vary for different materials.^4^, ^5^, ^6^, ^7^, ^8^, ^9^, ^10^, ^11^, ^12^, ^13^, ^14^, ^15^, ^16^, ^17^, ^18^, ^19^, ^20^, ^21^, ^22^, ^23^, ^24^, ^25^, ^26^, ^27^, ^28^, ^29^ Also, the technique can be used when limited amounts of test substances are available or if the test substance is difficult to aerosolize. It is faster (more cost‐effective) than inhalation exposure and hence allows for investigation of more substances. In the case of highly toxic, carcinogenic, or radioactive compounds, the potential risk for the personnel handling of the substances or the animals is also reduced.^30^ Another aspect is that a single intratracheal instillation may be less stressful for the animals compared with a long restrained inhalation exposure.^31^ We have previously demonstrated, using a variety of techniques, that nanomaterial dosed by intratracheal instillation is widely and evenly distributed throughout the lung,^32^ and that carbon nanotubes dosed by intratracheal instillation reach all lobes of the lung.^28^ Furthermore, two different carbon nanotubes, NM‐401 and NM‐403, dosed by inhalation or by intratracheal instillation induced dose‐dependent inflammation, where data from instillation and inhalation exposures fitted the same dose–response relationship curve^33^; similar effects of intratracheal instillation and inhalation were also seen for molybdenum disulfide and tungsten particles.^34^, ^35^, ^36^

Generally, intratracheal instillation reproduces the effects of inhalation. However, the bolus administration might produce a less homogeneous material distribution, with more focal exposure than is seen after inhalation exposure. In addition, intratracheal instillation forces the material into the alveoli, resulting in lower deposition in bronchia or bronchioles and it may overwhelm mucociliary clearance.^37^ The intratracheal instillation procedure involves direct deposition of materials into the trachea by cannulation of the larynx with a catheter, through the laryngeal folds, via which liquid can be injected into the proximal trachea, in anesthetized mice. The procedure itself induces low‐grade inflammation.

The instillation procedure implies that the particles under study are suspended in a vehicle. We have previously compared different vehicles,^38^ and found that pure water or 2% serum in water are the preferred vehicles.

In the current work, we wanted to assess whether the low‐grade inflammation induced by the instillation procedure can be minimized. Intratracheal instillation induced low levels of inflammation 24 h post‐exposure in vehicle‐exposed controls compared to inhalation exposure where controls inhaled filtered air.^37^, ^39^, ^40^, ^41^ The study by Jacobsen et al. compared inhalation and instillation exposure to different nanomaterials, and it was shown that there were 5.3 ± 1.6% neutrophils in bronchoalveolar lavage (BAL) cells in control mice exposed by intratracheal instillation compared to 1.1 ± 0.4% neutrophils in BAL cells following inhalation exposure.^37^ The highest exposure‐related background inflammation in terms of neutrophil influx has in many studies been demonstrated to occur 1 day post‐exposure; and lower neutrophil numbers are in general observed in vehicle‐exposed mice 3, 28 and 90 days post‐exposure.^6^, ^7^, ^9^, ^10^, ^24^, ^42^, ^43^

When measuring the effects of substances, the control/vehicle‐induced inflammation should be as low as possible, that is, similar to unexposed mice, to have optimal conditions for measuring substance‐induced inflammation. Thus, it is desirable to optimize the procedure in this respect. For intratracheal instillation, it may be an advantage to use disposable syringes to minimize the risk of cross‐contamination from reusable exposure devices, as, even if the devices can be cleaned and autoclaved prior to re‐use, there is a slight potential for retention of residual test material and oral flora. We therefore tested whether two different instillation devices, namely a reusable 250 μL reusable glass syringe/use‐once Insyte catheter device (Glass‐syringe/plastic‐catheter) and a use‐once 1‐mL syringe/reusable ball‐tipped steel‐needle (Disposable‐syringe/steel‐needle), induced different levels of inflammation in the lungs following intratracheal instillation. In addition, during the instillation, the anesthetized mice may still have chewed feed pellets stored in the cheek, which may translocate to the lungs during instillation causing airway inflammation. One solution would be to remove feed for a period prior to exposure. Another alternative would be to provide liquid feed before instillation exposure. We have tested the exposure‐related inflammation following intratracheal exposure to pure water using two different instillation device combinations in mice fed either pellets or liquid feed.

## 2 METHODS

### 2.1 Experiment 1—Comparison of untreated animals and intratracheally instilled animals

The animal procedures complied with the EC Directive 86/609/EEC and Danish law regulating experiments with animals (The Danish Ministry of Justice, Animal Experiments Inspectorate permission 2015−15−0201−00465), and were approved by the local animal ethical committee. Female C57BL/6NTac were obtained from Taconic (TAC mice, body weight 20 g, age 8 weeks; *n* = 19). Female C57BL/6NRj were obtained from Janvier Labs (body weight 20 g, age 8 weeks; *n* = 19). The mice were housed six per cage in polypropylene cages with Tapwai bedding and Enviro‐Dri enrichment (Brogaarden, Gentofte, Denmark). Wood blocks (Brogaarden, Gentofte, Denmark) and Hides (Mouse House, Scanbur, Karlslunde, Denmark) were placed in the cages as supplemental enrichment. The mice were given ad libitum access to pelleted feed (Altromin no. 1324, Christian Petersen, Denmark) and tap water. The room temperature was 20 ± 2°C, and humidity was 50 ± 20%. The animals were kept under a 12 h light: 12 h dark cycle (from 6 a.m. to 6 p.m.). The mice arrived 1 week before the start of the experiment to allow for acclimatization before the experiment was undertaken.

**FIGURE 1:**
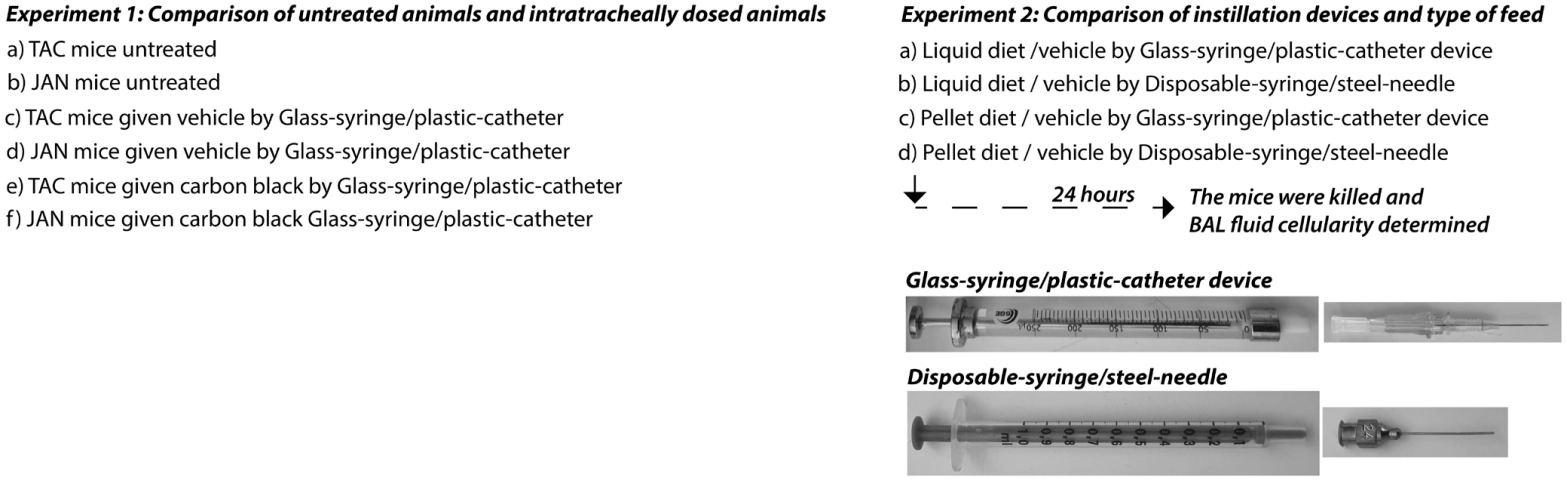
Illustration of the experimental set‐up and the two intratracheal instillation device combinations. The change to a liquid diet was undertaken 48 h before intratracheal instillation.

For each mouse strain, 5 mice were untreated, 6 were intratracheally instilled by Glass‐syringe/plastic‐catheter with vehicle (2% serum from other mice from the same inbred strain) only, and 6 mice were instilled by Glass‐syringe/plastic‐catheter with Printex 90 carbon black nanoparticles as a benchmark particle (54 μg per mouse instilled in 50 μL Nanopure water (18.2 MΩ ionic purity) with 2% serum). After 24 h of post‐exposure recovery, the animals were killed and BAL fluid was taken. An overview of the groups and the instillation device is provided in Figure 1.

### 2.2 Experiment 2—Comparison of instillation devices and type of feed

The study was made under the same Animal Experiments Inspectorate permission as the above‐described experiment (permission number: 2015−15−0201−00465) and complied with the EC Directive 86/609/EEC on the use of experimental animals. Female C57BL/6J mice, 7 weeks of age were obtained from Janvier (France; JAN mice). The mice were randomly distributed to the cages and housed 5 per cage. Each group contained 10 animals (*n* = 40 in total). The mice were housed in polypropylene cages with Tapwai bedding and Enviro‐Dri enrichment (Brogaarden, Gentofte, Denmark). Wood blocks (Brogaarden, Gentofte, Denmark) and Hides (Mouse House, Scanbur, Karlslunde, Denmark) served as further enrichment. The mice were given ad libitum access to feed (Altromin no. 1324—changed to liquid feed in two groups as described below; Christian Petersen, Denmark) and tap water. The room temperature was 20 ± 2°C, and humidity was 50 ± 20%. The animals were kept under a 12 h light: 12 h dark cycle (from 6 a.m. to 6 p.m.). The mice arrived 1 week before the start of the experiment to allow for acclimatization before the experiment was undertaken.

#### 2.2.1 Intratracheal instillation device combinations tested in Experiment 2

The mice were intratracheally instillated with one of two device combinations. The experimental set‐up and the device combinations are illustrated in Figure 1. The first combination had a 250 μL glass syringe with a steel plunger (SGE Syringe, Model 250F‐LT‐GT from Trajan Scientific and Medical: Fixed Luer Tip, Supplier number: 006229, VWR Catalog Number 60361‐436, VWR Denmark). This syringe was combined with an Insyte catheter (BD Insyte 22GA 1.00IN, 0.9 × 25 mm) without a needle (abbreviated: Glass‐syringe/plastic‐catheter). This is a commonly used combination and the standard procedure in our laboratory.^30^ The glass syringe is cleaned and sterilized by autoclaving prior to use.

The second device combination had a 1 mL disposable syringe with a plastic plunger (B.Braun Injekt®–F Solo, 1 mL without a cannula, Luer connector) the cylinder material was polypropylene, the piston material was polyethylene. The sterile syringe was silicone‐, latex‐ and PVC‐free. The syringes were individually sterile‐packed and manufactured according to EN ISO 7886‐1. This syringe was combined with a ball‐tipped stainless steel feeding needle (AgnTho's 7900 Feeding needles, straight, 24G 1,25 mm tip, 25 mm, AgnTho's AB, Sweden). The second device combination is termed Disposable‐syringe/steel‐needle.

#### 2.2.2 Instillation vehicle and types of feed tested

The instillation vehicle was Nanopure water (50 μL). Forty‐eight hours before intratracheal instillation, the feed was changed to a liquid diet for two groups, while the remaining two groups continued on pelleted feed with the same nutrient composition (Altromin 1324) (Figure 1). The liquid feed was made with 1 volume of powder and 2 volumes of water (Altromin 1321 composition similar to Altromin 1324). The content of multiple elements in the diets is provided in Table S1 to provide an overview of trace metals that could be speculated to contribute to a potential excessive inflammation.

### 2.3 Detailed description of the intratracheal instillation procedure

For the intratracheal instillation procedure, the mice were anesthetized by isoflurane and then placed lying on its back on a 40‐degree slope. Next, a diode light was placed in contact with the larynx, and the tongue of the mice was pressed against the lower jaw with of a spatula. Then the trachea was intubated either with the 24‐gauge BD Insyte catheter, modified so the needle was cut about 2 mm shorter than the silicone catheter, or, with the reusable metal needle in the alternative device combination. Whether the intubation was placed correctly was tested with a small but highly sensitive pressure transducer (developed by the National Research Centre for the Working Environment in collaboration with John Frederiksen; FFE/P, Copenhagen, Denmark). Correct intubation is visually shown on a meter following the variation in pressure caused by inspiration and expiration. Next, the instillation was performed by placement of the syringe (either the 250 μL glass onto the Insyte catheter; Glass‐syringe/plastic‐catheter) or the disposable 1 mL syringe onto the ball‐tipped steel‐needle (Disposable‐syringe/steel‐needle). The 50 μL vehicle solution was then instilled and, in one go, immediately followed by the injection of 200 μL of air (placed in the syringe above the liquid). The animal was then taken off the 40‐degree slope, held with the head up, and twice lowered in a 10–15‐cm quick descending movement—intended to distribute the liquid into the lungs and to prevent blocking of the airways.^37^ Afterward, the animal was placed in its cage, which was placed on a 37°C heating plate. Half of the mice were instilled on 1 day, the other half were instilled 1 week later. The mice were killed 24 h after instillation by subcutaneous injection of Zoletil 100 (Zolazepam 250 mg/mL and Tiletamine 250 mg/mL), Xysol (Xylazine 20 mg/mL), and Fentadon (Fentanyl 50 μg/mL) in sterile saline, at a volume of 0.1 mL/10 g bw, followed by exsanguination without prior cervical dislocation.

The glass syringes and steel ball tips were cleaned before use by, first, a thorough wash with 2% SDS in water, then a thorough rinse with saline 0.9% and finally a wash with acetone before autoclavation. Separate instruments were used for each treatment group, that is, all animals in a group were instilled with the same needle and tip with no cleaning between animals.

### 2.4 Humane endpoint monitoring during the studies

Body weight loss was monitored, with a 10% decrease in body weight resulting in increased attention to the mice. At 15%, a red flag is raised for the condition of the particular mouse, as a 20% loss in body weight defines the humane threshold where excessive toxicity warrants immediate euthanasia. Other humane endpoints monitored were clinical appearance, by examination of body temperature, raised fur, grimace pain scale, natural behavior, and activity level. One of the indicators of toxicity is inactivity, even when being grabbed or handled.

### 2.5 Determination of BAL fluid cellularity

BAL fluid was recovered after exsanguination as previously described.^38^ Briefly, the collection of BAL fluid was done after exsanguination of the animals, by flushing the lungs with 0.9% sodium chloride solution. In preparation for the procedure, the skin was cut open to expose the larynx, and in the cranial part of the trachea, a small incision was made for a customized 1 mL syringe with a 22 gauge needle mounted with ~2 cm plastic tube (Intramedic Clay Adams brand propylene tubing with inner diameter 0.58 mm and outer diameter 0.965 mm). Then, 0.8 mL of the NaCl solution was injected into the lung and thereafter redrawn into the syringe. This procedure was repeated with a new volume of solution, and the two obtained volumes were combined in a single vial. Next, the BAL fluid was centrifuged at 400×*g* for 10 min at 4°C to isolate the cells from the fluid. The supernatants were recovered and frozen for potential biochemical measurements. The cells were re‐suspended in 100 μL of HAMF12 medium with 10% fetal bovine serum (Prod numbers: 217654037 and 10 106 169, Invitrogen, Carlsbad, CA, USA). For differential counting of the BAL cells, a volume of 40 μL of the cell suspension was transferred to a Cytofuge 2 holder and spun at 1000 RPM for 4 min (StatSpin, Bie and Berntsen, Rødovre, Denmark). The slides were then left to dry for at least 30 min, after which the cells were fixed with 96% ethanol for 5 min and stained with May‐Grünwald‐Giemsa stain. Differential counting was done on 200 cells by determining the number of neutrophils along with other cell types. The total number of neutrophils in the BAL was calculated by adjusting to the total cell numbers determined in fresh BAL fluid on a NucleoCounter NC‐100 (Chemometec, Allerød, Denmark).

### 2.6 Statistical analysis

**FIGURE 2:**
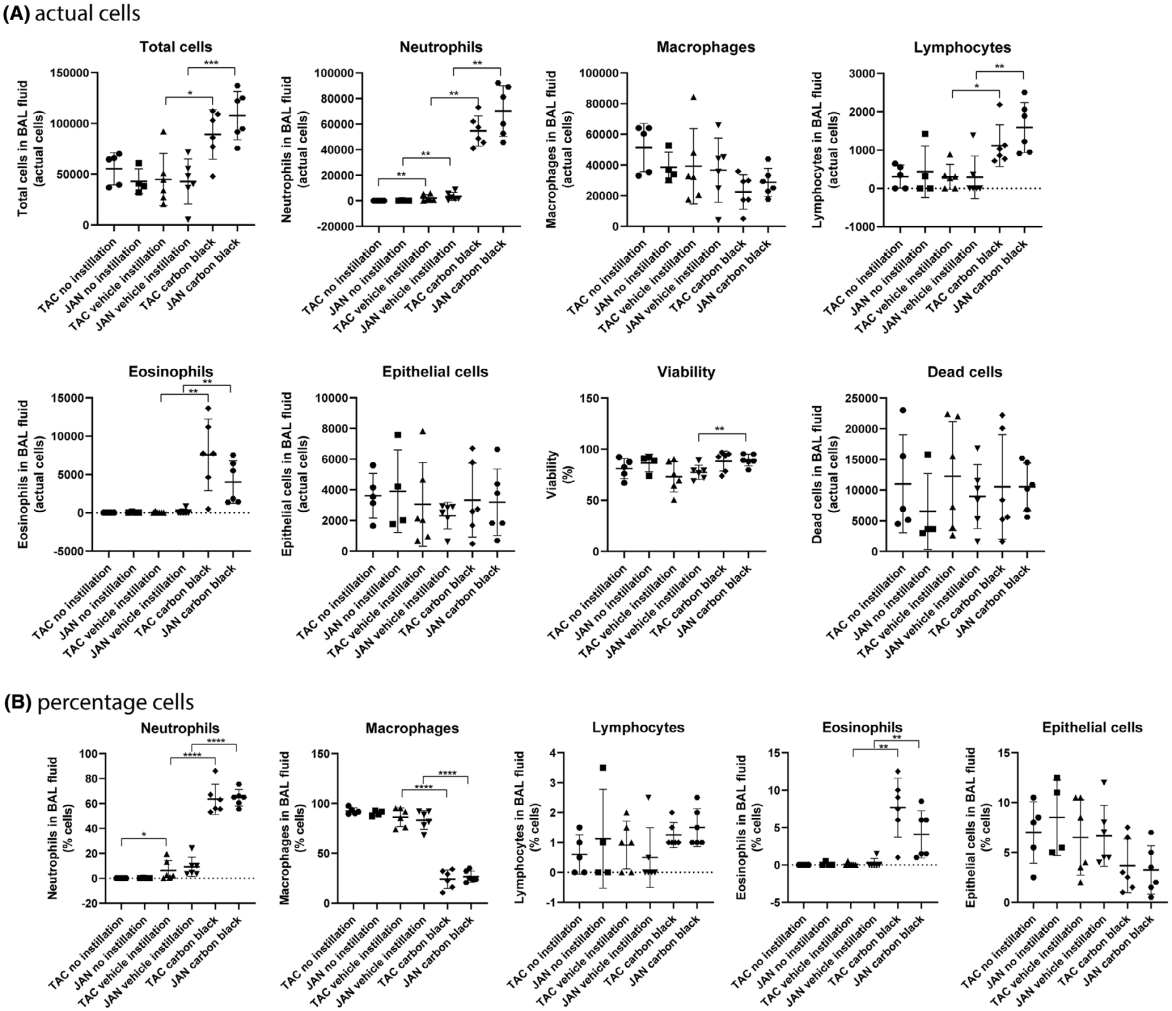
BAL fluid cellularity of untreated animals, vehicle or carbon black instilled. The number of mice in each groups was 5 for untreated and 6 in the other 4 groups. The upper panel (A) shows data expressed as actual cells; the lower panel (B) shows data expressed as the percentage of total cells in the BAL. TAC, mice from Taconic; JAN, of mice from Janvier. Data are mean and bars represent SD. ****, ***, **, and * designate *t*‐test (or the Mann–Whitney test) *p* values of <0.0001, <0.001, <0.01 and <0.05, respectively, versus the group indicated by the horizontal bar placed above the columns.

**FIGURE 3:**
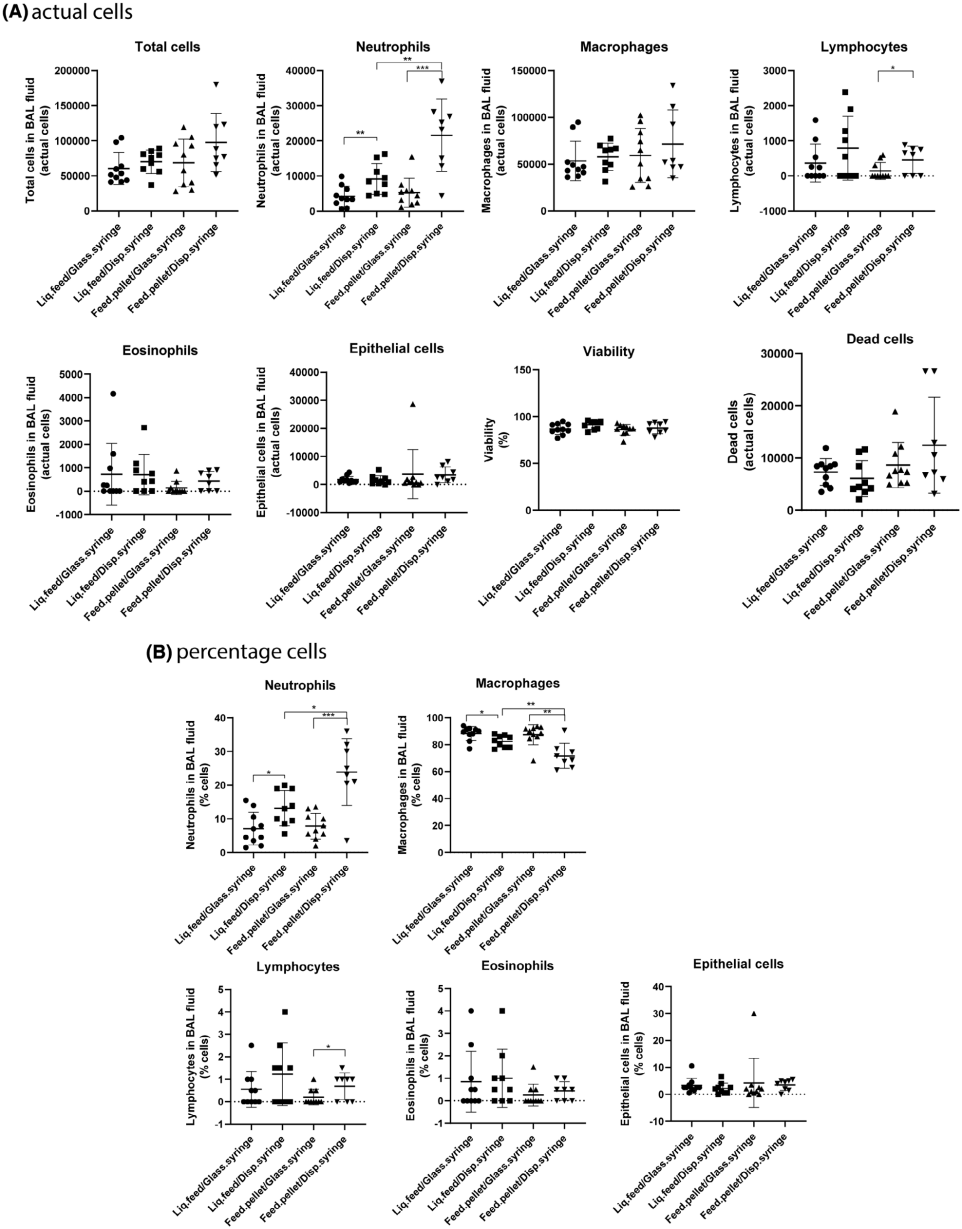
BAL fluid cellularity in the experiment comparing different instillation device and feed combinations. The mice were from Janvier (France). Each group contained 10 animals. The upper panel (A) shows data expressed as actual cells; the lower panel (B) shows data expressed as the percentage of total cells in the BAL fluid. Liq.feed/Glass.syringe, liquid feed/Glass‐syringe/plastic‐catheter; Liq.feed/Disp.syringe, liquid feed/Disposable‐syringe/steel‐needle; Feed.pellet/Glass.syringe, feed pellets/Glass‐syringe/plastic‐catheter; Feed.pellet/Disp.syringe, feed pellets/Disposable‐syringe/steel‐needle. Data are mean and bars represent SD. ***, **, and * designate *p* values of <0.001, <0.01 and <0.05, respectively, of the *t*‐test (or the Mann–Whitney test) versus the group indicated by the horizontal bar placed above the columns.

Statistical analyses were performed using the Graph Pad Prism 8.02 software package (Graph Pad Software Inc., La Jolla, CA, USA). Data were initially tested for normality using the Shapiro–Wilk test. We considered the *t* test a robust measure of deviations from normality and we thus employed it for intergroup comparisons except if the *p* value of the Shapiro Wilk test was very low (*p* < 0.001) Another exception was when the standard deviations of the groups were very different as determined with the *F* test (*p* < 0.001). In these cases, we used the non‐parametric Mann–Whitney test. No Bonferroni correction was employed and a *p* value of <0.05 was considered significant. The following comparisons were performed for each individual cell type and for the total number of cells: (Figure 2) vehicle vs. no instillation (both mouse strains), vehicle vs. carbon black (both mouse strains); (Figure 3): liquid feed/glass syringe versus liquid feed/disposable syringe, feed pellets/glass syringe versus feed pellets/disposable syringe, liquid feed/glass syringe versus feed pellets/glass syringe, and liquid feed/ disposable syringe versus feed pellets/disposable syringe.

## 3 RESULTS

### 3.1 Comparison of untreated and intratracheally instilled animals

Intratracheal instillation of vehicle was assessed using 2% serum in water as a preferred vehicle for nanomaterials.^38^ The vehicle induced a slight increase in neutrophil counts 24 h after exposure compared to untreated mice (Figure 2, upper panel). The increase was small compared to the increase in neutrophils following instillation of the benchmark particle, Printex 90 carbon black nanoparticles. None of the other cell types (macrophages, lymphocytes, eosinophils, and epithelial cells) differed between untreated animals and vehicle‐instilled animals, while for total cells, lymphocytes, and eosinophils, carbon black caused increases (Figure 2). Pulmonary exposure to 162 μg carbon black induced similar neutrophil influxes in TAC and JAN mice. If the cell counts were presented as percentages of total cells in BAL fluid, the results were similar for neutrophils and eosinophils, while macrophages were decreased with carbon black (Figure 2, lower panel). Similar responses were observed in female C57BL/6NTac mice (Taconic) and female C57BL/6NRj mice (Janvier Labs) (Tabulated values are provided in Table S2).

### 3.2 Comparison of instillation devices and type of feed

We next assessed whether the type of instillation device and the type of feed influenced the procedure‐induced inflammation. Neutrophils (actual numbers) increased in both the liquid and pellet fed groups with use of the Disposable‐syringe/steel‐needle compared to the Glass‐syringe/plastic‐catheter combination. Moreover, with the Disposable‐syringe/steel‐needle, neutrophil influx was higher in the group fed pelleted feed compared with the liquid feed group (Figures 2 and 3). Lymphocytes were increased only in the pellet fed–disposable syringe/needle group in comparison to the pellet fed–Glass‐syringe/plastic‐catheter group (Figures 2 and 3). All other cell types, as well as total cells, viability, and the number of dead cells in BAL fluid, were not different among the four groups (Figures 2 and 3). When looking at the percentage of cells in BAL fluid, we saw the same pattern for neutrophils and lymphocytes as described above, while macrophages were affected in the same way as neutrophils except that macrophages decreased when neutrophils increased (Figure 3, lower panel). (Tabulated values are provided in Table S3).

## 4 DISCUSSION

### 4.1 Comparison on untreated animals and intratracheally instilled animals

Intratracheal instillation with vehicle (2% serum) induced slight inflammation compared to untreated female mice (Figure 3, upper panel). The increase was small compared to the increase in neutrophil levels following instillation of the benchmark particle, carbon black nanoparticles. Moreover, identical responses were seen in TAC and JAN mice. In the present study, we only included female mice for logistical reasons, since female mice are less aggressive than male mice and thus can be housed in groups of 5–6. We note that carbon black nanoparticle‐induced pulmonary inflammation is well documented in both male and female mice and rats.^44^, ^45^, ^46^, ^47^, ^48^, ^49^, ^50^ Thus, there is no reason to assume that male mice would display a different response.

In a previous study, we exposed male mice by intratracheal instillation to pure water or different nanomaterials suspended in pure water. The mice were exposed once weekly for 7 weeks and killed 6–8 days after final exposure.^51^ In that study, untreated mice were included, allowing for comparison between unexposed and vehicle‐exposed mice. For mice exposed to pure water, BAL cell counts showed 4000 ± 2000 neutrophils corresponding to 0.37% of the BAL cells. For untreated mice, there were 11 000 ± 2000 neutrophils, corresponding to 0.88% of the total BAL cells. Thus, 6–8 days after intratracheal instillation, a potential increase in inflammation resulting from the instillation procedure had returned to the level seen in untreated mice. In a study by Jackson et al. establishing an exposure protocol for the investigation of maternal developmental toxicity, the intratracheal instillation procedure done four times over 2 weeks did not affect maternal body weight nor were there any differences in BAL fluid cell composition, indicating that the procedure did not cause detectable inflammation 3 days after the last exposure.^30^

We have previously assessed the effect of vehicle choice on inflammation in vehicle‐exposed mice. Rats and mice were exposed to different vehicles (instillation dispersion media) by intratracheal instillation with or without different nanomaterials. In the control groups, the dispersion media were 2% serum in water, 0.05% serum albumin in water, 10% bronchoalveolar lavage fluid in 0.9% NaCl, 10% bronchoalveolar lavage fluid in water, or 0.1% Tween‐80 in water. In terms of neutrophil numbers in BAL fluid, there was no difference between the various vehicles in these control groups without nanomaterials. Yet, in terms of the comet assay, the 2% serum showed a slightly lower level of DNA damage in BAL fluid cells from control mice compared to control mice given either water or 0.05% BSA in water.^52^ As 2% serum in water or pure water are the preferred vehicles in our studies, we used this vehicle in the present study.

### 4.2 Comparison of instillation devices and type of feed

When assessing the effect of instillation devices and type of feed, the Glass‐syringe/plastic‐catheter combination caused lower inflammation than the Disposable‐syringe/steel‐needle combination. We have no explanation for this difference. It seems unlikely that any substances in the material makeup of the disposable syringes is responsible for the higher inflammation. We note that it may be more difficult to provide the correct dose with the plastic syringe made for medical use compared to the reusable glass syringes made for analytical chemistry—that is, the dose indications on the syringes may be more precise on the latter, but this would rather cause larger variation in substance‐instilled groups than in the vehicle‐only groups.

Neutrophil influx was higher for the groups exposed using the disposable syringe/steel needle compared to the group exposed using the Hamilton syringe. The highest neutrophil response was observed in the group exposed using disposable syringe/steel needle and fed pellets. The increased neutrophil influx was seen in most of the animals. There are many possible reasons for this exposure‐related inflammation, including that a slight scratching of the throat by the disposable syringe in combination with co‐exposure to food debris stored in the cheek or microorganisms from the feed could contribute to the slightly increased inflammatory response. However, the instillation‐related neutrophil influx is still much lower than the neutrophil influx induced by exposure to carbon black nanoparticles.

## 5 CONCLUSION

We found that the use of a 250 μL sterilized reusable glass syringe with an Insyte plastic catheter (Glass‐syringe/plastic‐catheter) for intratracheal instillation in mice caused less inflammation than a disposable 1 mL‐syringe with ball‐tipped steel‐needle (Disposable‐syringe/steel‐needle). Moreover, when using the Glass‐syringe/plastic‐catheter device there was no advantage to using liquid feed over feed pellets. These findings confirm the Glass‐syringe/plastic‐catheter combination as a preferred instillation procedure.

## AUTHOR CONTRIBUTIONS

**Niels Hadrup:** Formal analysis; visualization; writing – original draft. **Michael Guldbrandsen:** Conceptualization; investigation; writing – review and editing. **Eva Terrida:** Conceptualization; investigation; writing – original draft. **Katja M. S. Bendtsen:** Conceptualization; investigation; writing – review and editing. **Karin S. Hougaard:** Methodology; writing – review and editing. **Nicklas R. Jacobsen:** Methodology; writing – review and editing. **Ulla Vogel:** Conceptualization; formal analysis; writing – original draft.

## FUNDING INFORMATION

This work was supported by the Focused Research Effort on Chemicals in the Working Environment (FFIKA) from the Danish Government.

## CONFLICT OF INTEREST STATEMENT

The authors report there are no competing interests to declare.

## ETHICS STATEMENT

The animal procedures complied with the EC Directive 86/609/EEC and Danish law regulating experiments with animals (The Danish Ministry of Justice, Animal Experiments Inspectorate permission 2015−15−0201−00465) and were approved by the local animal ethical committee.

## Supporting information


Data S1:

## Data Availability

The data are available upon request to the authors.
