# Supplementary material for: Intratracheal instillation for the testing of pulmonary toxicity in mice—Effects of instillation devices and feed type on inflammation
Source: Animal Model Exp Med. 2025 Jan 3;8(2):378–86. doi: 10.1002/ame2.12503 (PMC11871123; doi:10.1002/ame2.12503)
Supplement: Supplementary file 1 — Data S1: [file AME2-8-378-s001.docx]

**Supplemental Materials Hadrup et al.**

**Table S1 Concentration of elements in Altromin 1324 standard diet.** Taken from (Altromin, 2023). Notably the liquid diet is made from the same formula just as a powder called Altromin 1321. Collectively the formula is called Altromin 1320.

| **Minerals** | **Concentration (% of the diet)** |
| --- | --- |
| Calcium | 0.7 |
| Potassium | 0.9 |
| Magnesium | 0.2 |
| Sodium | 0.2 |
| Phosphorus | 0.5 |
| **Trace elements** | **Concentrations (mg/kg diet)** |
| Aluminium | 79.4 |
| Chlorine | 3,484.1 |
| Iron | 192.5 |
| Fluorine | 2.8 |
| Iodine | 1.7 |
| Cobalt | 0.34 |
| Copper | 12.8 |
| Manganese | 95.1 |
| Molybdenum | 1.1 |
| Sulphur | 1,141.2 |
| Selenium | 0.25 |
| Zinc | 95.2 |

**Table S2. BAL fluid cellularity of animals that were not instillated and animals that were instillated with either vehicle or carbon black**

|  | Neutrophils |  | Macrophages |  | Lymphocytes |  | Eosinophils |  | Epithelial cells |  |
| --- | --- | --- | --- | --- | --- | --- | --- | --- | --- | --- |
|  | Mean | SD | Mean | SD | Mean | SD | Mean | SD | Mean | SD |
| TAC no instillation | 0 | 0 | 51415 | 15780 | 310 | 302 | 0 | 0 | 3615 | 1447 |
| Jan no instillation | 76 | 152 | 38453 | 10004 | 435 | 672 | 40 | 81 | 3896 | 2692 |
| TAC vehicle instillation | 2122 | 2646 | 39243 | 24590 | 301 | 325 | 37 | 91 | 3047 | 2733 |
| Jan vehicle instillation | 3468 | 3093 | 36617 | 20938 | 290 | 554 | 138 | 339 | 2313 | 878 |
| TAC carbon black instillation | 54669 | 11897 | 22450 | 11271 | 1113 | 545 | 7563 | 4667 | 3322 | 2412 |
| JAN carbon black instillation | 70182 | 19821 | 28670 | 9136 | 1588 | 654 | 4005 | 2778 | 3189 | 2172 |

|  | Total cells |  | Viability (%) |  | Dead cells |  |
| --- | --- | --- | --- | --- | --- | --- |
|  | Mean | SD | Mean | SD | Mean | SD |
| TAC no instillation | 55340 | 15857 | 81 | 10 | 11018 | 8017 |
| Jan no instillation | 42900 | 12372 | 87 | 9 | 6500 | 6208 |
| TAC vehicle instillation | 44750 | 25703 | 73 | 15 | 12287 | 8890 |
| Jan vehicle instillation | 42827 | 22276 | 77 | 7 | 8960 | 5244 |
| TAC carbon black instillation | 89117 | 24397 | 88 | 10 | 10525 | 8533 |
| JAN carbon black instillation | 107633 | 23782 | 89 | 6 | 10528 | 3906 |

**Table S3. BAL fluid cellularity in the experiment of different instillation device combinations**

|  | Neutrophils |  | Macrophages |  | Lymphocytes |  | Eosinophils |  | Epithelial cells |  |
| --- | --- | --- | --- | --- | --- | --- | --- | --- | --- | --- |
|  | Mean | SD | Mean | SD | Mean | SD | Mean | SD | Mean | SD |
| Liquid food / Hamilton | 4215 | 2916 | 53449 | 20978 | 366 | 546 | 723 | 1321 | 1737 | 1219 |
| Liquid food/ Disposable syringe | 9169 | 4394 | 57802 | 14614 | 793 | 909 | 709 | 864 | 1594 | 1606 |
| Food Pellets/Hamilton | 5293 | 4099 | 59354 | 29003 | 142 | 240 | 141 | 285 | 3691 | 8806 |
| Food pellets/Disposable syringe | 21580 | 10285 | 71611 | 36154 | 457 | 387 | 437 | 404 | 3427 | 2800 |

|  | Total cells |  | Viability (%) |  | Dead cells |  |
| --- | --- | --- | --- | --- | --- | --- |
|  | Mean | SD | Mean | SD | Mean | SD |
| Liquid food / Hamilton | 60490 | 22684 | 87 | 6 | 7315 | 2564 |
| Liquid food/ Disposable syringe | 70067 | 16772 | 91 | 5 | 6090 | 3452 |
| Food Pellets/Hamilton | 68620 | 33913 | 86 | 6 | 8654 | 4311 |
| Food pellets/Disposable syringe | 60490 | 41620 | 88 | 6 | 12444 | 9215 |
